# Prediction and Analysis of Surface Hydrophobic Residues in Tertiary Structure of Proteins

**DOI:** 10.1155/2014/971258

**Published:** 2014-01-09

**Authors:** Shambhu Malleshappa Gowder, Jhinuk Chatterjee, Tanusree Chaudhuri, Kusum Paul

**Affiliations:** ^1^Department of Biotechnology, The Oxford College of Engineering, Bangalore 560068, India; ^2^Department of Biotechnology, PES Institute of Technology, Bangalore 560085, India

## Abstract

The analysis of protein structures provides plenty of information about the factors governing the folding and stability of proteins, the preferred amino acids in the protein environment, the location of the residues in the interior/surface of a protein and so forth. In general, hydrophobic residues such as Val, Leu, Ile, Phe, and Met tend to be buried in the interior and polar side chains exposed to solvent. The present work depends on sequence as well as structural information of the protein and aims to understand nature of hydrophobic residues on the protein surfaces. It is based on the nonredundant data set of 218 monomeric proteins. Solvent accessibility of each protein was determined using NACCESS software and then obtained the homologous sequences to understand how well solvent exposed and buried hydrophobic residues are evolutionarily conserved and assigned the confidence scores to hydrophobic residues to be buried or solvent exposed based on the information obtained from conservation score and knowledge of flanking regions of hydrophobic residues. In the absence of a three-dimensional structure, the ability to predict surface accessibility of hydrophobic residues directly from the sequence is of great help in choosing the sites of chemical modification or specific mutations and in the studies of protein stability and molecular interactions.

## 1. Introduction

Knowledge of protein stability is crucial for understanding of the basic thermodynamics of the process of folding. The hydrophobic effect is considered to be the major driving force for the folding of globular proteins [1]. The hydrophobic effect is driven by the entropy increase of the solvent water molecules; hydrophobic side chains are located predominantly in the interior of a protein. This arrangement stabilizes the folded polypeptide backbone, since unfolding it or extending it would expose the hydrophobic side chains to the solvent. The hydrophobicity analysis has remained at the central focus for understanding protein folding and stability. It has been hypothesized that hydrophobic interactions play a major role in organizing and stabilizing the architecture of proteins [2]. As their name implies, hydrophobic amino acids have essentially nonpolar side chains, for example, valine, leucine, isoleucine, phenylalanine, and methionine fit into this group. In proteins, hydrophobic residues tend to be buried in the interior of the protein away from the solvent and polar side chains are exposed to the solvent. The folding process of polypeptide chain depends on the hydrophobicity of the side chains. It is now widely accepted that hydrophobicity is a dominant force of protein folding [3, 4]. There is a linear relationship between the surface areas of amino acid residues (in a standard state) and the free energy changes associated with the transfer of the amino acids from water to organic solvent [5–7].

One strategy to increase the stability of proteins is to reduce the area of water-accessible hydrophobic surface [8].

Solvent accessibility plays an important role in the structure and functions of biological macromolecules. Generally amino acid residues located on the surface of a protein serve as active sites and/or interact with other molecules and ligands [9]. The concept of solvent accessibility is widely used to understand the location of amino acid residues in protein structures and their contribution to the stability of the protein.

The folding process of soluble proteins decreases the surface in contact with the solvent. This is related to the secondary structures of proteins. Accurate knowledge of residue accessibility would thus aid the prediction of secondary structures. Different methods of prediction are based on the use of protein structure databases and on multiple sequence alignments. They have various efficiencies, notably, depending on the number of relative accessibility states that is, exposed, 2 buried, and in-between; [10–14].

The accessible surface area of the protein is calculable from a set of coordinates which measures the thermodynamic interaction between protein and water. Surface area accessibility calculations identify which residues are solvent exposed and which residues are buried, contributing to the hydrophobic stabilization of protein structure. In the case of the solvent accessibility prediction, using evolutionary information such as multiple sequence alignment and position-specific scoring matrix has generally given good prediction results [15]. From MSA (multiple sequence alignment), we analyzed how well solvent exposed and buried hydrophobic residues are evolutionarily conserved on the nonredundant data set of 218 monomeric proteins.

## 2. Materials and Methods

### 2.1. Data Set

In the present study, total of 4154 monomeric proteins were obtained from PIQSI (quaternary structure database) [16]. We have filtered out those proteins to get nonredundant monomeric proteins dataset from PDB (protein data bank) [17] which has the following features: (i) X-ray resolution less than 2 Å for better resolution, (ii) percentage of similarity cut-off less than 30%, (iii) having a biological assembly unit, and (iv) chain length not less than 50 residues, and finally nonredundant datasets of 218 proteins were obtained.

### 2.2. Computation of Solvent Access Surface Area

The ASA (accessible surface area) is defined as the locus of the center of the solvent molecule as it rolls over van der Waals surface of the protein [7]. The software NACCESS [18] was used to calculate ASA for all atoms in PDB file. The ASA is calculated using Lee-Richards (1971) formula [19], whereby a probe of a given radius is rolled around the surface of the molecule, and the path traced out by its center is the accessible surface:

(1) $$\begin{array}{c} \text{ASA}=\sum \left[\frac{R}{{\left(R^{2}-Z_{i}^{2}\right)}^{1/2}}\right]L_{i}\cdot D;\\ D=\frac{\Delta Z}{2}+\Delta^{\prime }Z, \end{array}$$

where *L*_*i*_ is the length of the arc computed on a given section *I*, *Z*_*i*_ is the perpendicular distance from the center of the sphere to the section *I*, Δ*Z* is the spacing between the sections, and Δ′*Z* is Δ*Z*/2 or *R* − *Z*_*i*_, whichever is smaller.

### 2.3. Relative Solvent Accessibility

RSA (relative accessible surface area) is defined as the per residue ratio between ASA and references value for particular residue. RSA file containing summed atomic accessible surface areas over each protein or nucleic acid residue, as well as the relative accessibility of each residue calculated as the % accessibility compared to the accessibility of that residue type in an extended ALA-x-ALA tripeptide for amino acids [20].

The pictorial representation of such RSA values provides an easy understanding of the location of each residue in the structure of protein. It will also reveal the population of each residue on the surface and interior core of a protein.

Threshold to distinguish 2 states is also specified. We have classified residues based on threshold values of RSA cut-off used by Zhu and Blundell [21] If the RSA percentage is greater than 7, it will be considered as solvent exposed residue and RSA percentage is less than 7, it will be considered as buried residue.

### 2.4. Residue Propensity

During the process of protein folding, the amino acid residues along with the polypeptide chain interact with each other in a cooperative manner to form stable native structure and also form clusters. Zehfus reported that averages of 65% of hydrophobic residues are involved in residue clusters and each hydrophobic cluster contains at least five residues. Probably, hydrophobic residues (FMILYVW) occur frequently within buried area and flanking the gapped region [22, 23].

In order to analyze the hydrophobic cluster in proteins and to understand the influence of interresidue interactions to the formation of residue clusters, which are important for the folding and stability of protein structures, we have calculated propensity of each residue type on the surface and buried area in order to know each residue's natural tendency towards buried area and exposed area.

### 2.5. Propensity Calculation

The Following equations refer to propensity calculation towards surface and buried area:

(2) SURFACEPROPENSITY =(Total  no  of  solvent  exposed  specific  type  residuesTotal  no  of  solvent  exposed  residues)  ×(Total  no  of  specific  type  residuesTotal  no  of  residues)−1,

(3) BURIEDPROPENSITY =(Total  no  of  buried  specific  type  residuesTotal  no  of  buried  residues)  ×(Total  no  of  specific  type  residuesTotal  no  of  residues)−1.

Similarly as mentioned in (2) and (3), we have also calculated propensity of hydrophobic residues for flanking regions both for buried and exposed hydrophobic residues. (i) +1 and −1 (ii) +2 and −2 regions are considered for flanking residues.

### 2.6. Searching for Homologous Sequences for Each of 218 Monomer Proteins

By the nature of proteins, we know that solvent exposed hydrophobic residues are poorly conserved, but buried hydrophobic residues are highly conserved [24, 25]. In order to check the evolutionarily conserved hydrophobic residues on solvent exposed area and buried area, we used stand-alone BLASTP [26] for each individual protein against nonredundant dataset. Consider homologous sequences which have sequence identity greater than 30%.

### 2.7. Calculating Conservation Score Based on Hydrophobic Nature

Conservation score for all the residues in the protein can be obtained by comparing the sequence of a PDB chain with its respective homologous sequences using multiple sequence alignment. In our analysis, conservation score has been calculated based on hydrophobic nature evolutionarily in the alignment by applying following conditions:

1. in the alignment when any of these hydrophobic residues occur (Val, Ile, Leu, Met, and Phe) are scored 1;
2. similarly Ala and aromatic residues like Tyr and Trp that occur in the alignment are scored 0.5 because these three residues are partially hydrophobic and they tend to be buried and exposed equally;
3. if any polar residues occur in the alignment, then they are scored as −2 because they are hydrophilic in nature;
4. finally gap has been considered as −2 extra penalty is given for gap.

## 3. Results and Discussion

### 3.1. Interior and Surface Amino Acid Composition

**Figure 1:**
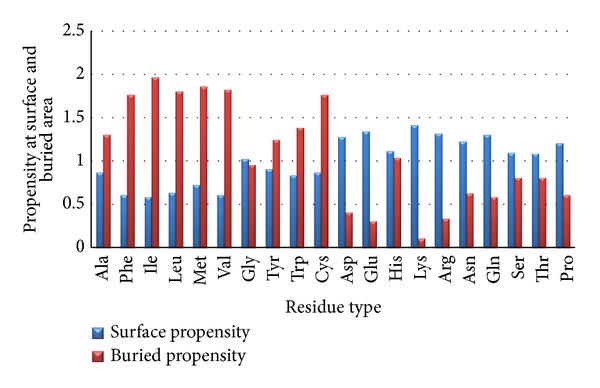
It refers to propensity of individual residues on surface and buried area.

To know the hydrophobic residues distribution in protein three dimensional structures we have performed structural analysis of 218 proteins using NACCESS server with respect to its RSA values, (details provided in supplementary file available online at http://dx.doi.org/10.1155/2014/971258) the following results were observed. 34.84% of hydrophobic residues occurred in total data set of proteins in which 77.1% of hydrophobic residues preferred in buried area and 22.9% of hydrophobic residues preferred in accessible surface area. Propensity of hydrophobic residues preference on protein surface and interior was calculated in order to analyze the hydrophobicity cluster (Figure 1).

The surface propensity and buried propensity for each residue described in the Figure 1 were calculated using (2) and (3), respectively. It has been observed from Figure 1 that large hydrophobic residues such as Val, Ile, Leu, Met, Phe, and including partial hydrophobic residues like Tyr, Try, and Ala have high propensity towards buried region compared to surface regions. Among hydrophobic residues, Ile has the highest propensity towards the buried region having a value of 1.96 and correspondingly Met has the highest tendency towards solvent exposed region (Figure 1).

Hydrophilic residues have high propensity towards surface region. Among all hydrophobic residues, His has a high tendency towards buried region and Lys has high tendency towards exposed region.

**Figure 2:**
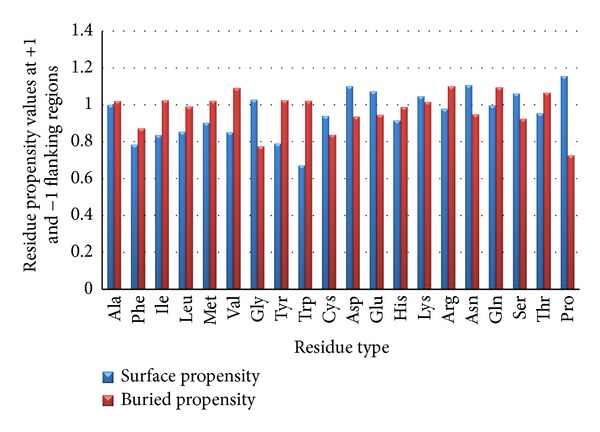
It refers to propensity of hydrophobic residues at +1 and −1 flanking position.

**Figure 3:**
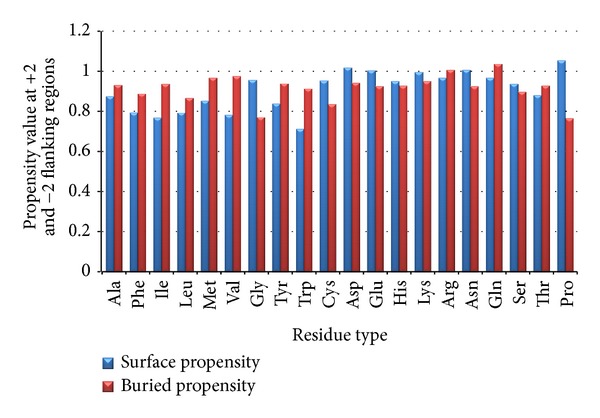
It refers to propensity of hydrophobic residues at +2 and −2 flanking position.

In order to analyze hydrophobic clusters appearing in surface or buried areas, flanking regions were considered. Figures 2 and 3 give a clear observation that hydrophobic cluster is more likely to come towards buried region than exposed region which is the range +1, −1 and +2, −2 present in the flanking region.

Significant changes for hydrophobic residues were not observed when flanking regions +1, −1 and +2, −2 were compared. The conservation score and knowledge of flanking regions of hydrophobic residues propensity towards buried and exposed area have been applied to the prediction of surface hydrophobic residues.

We needed to know how well surface and buried hydrophobic residues are conserved evolutionarily. Conservation score has been calculated for each residue of the query protein present in the complete data set using the knowledge of hydrophobic nature in the homologous sequences (Table 1).

**Figure 4:**
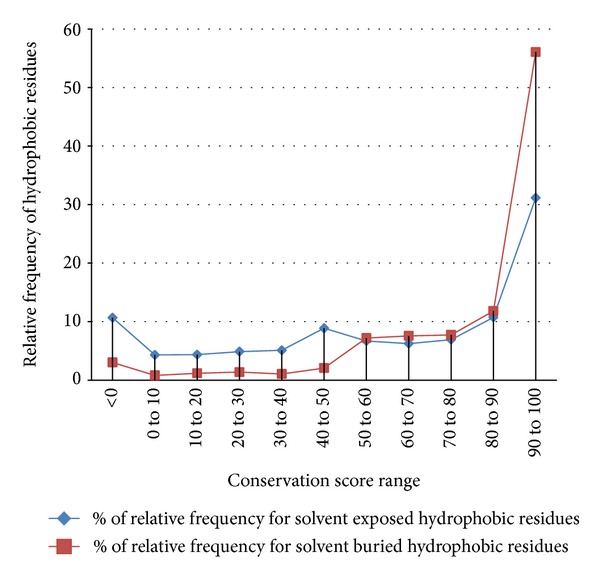
Relative frequency of hydrophobic residues versus conservation score range.

Figure 4 refers to relative frequency of solvent exposed and buried hydrophobic residues in respective conservative score bin.

As observed from Figure 4, solvent exposed hydrophobic residues are dominant than buried hydrophobic residues at conservation score range from 0 to 50. It has been observed that over 70% of exposed hydrophobic residues are falling in conservation score range from 0 to 50. Further, the observation leads to only 30% buried hydrophobic residues falling into the 0 to 50 conservation score ranges and remaining 70% falling into the 50 to 100 range. It shows that buried hydrophobic residues are highly conserved than exposed.

It has also been observed that at the conservation score range 60 to 70 there is an overlap, where in the buried hydrophobic residues start to take over exposed hydrophobic residues, they dominate in the conservation score range from 70 to 100.

### 3.2. Confidence Score Calculation

(a) Consider exposed hydrophobic residues

(4)  CONFIDENCE  SCORE=Solvent  exposed  hydrophobic  residues  normalized  score  at  the  range  [0  to  10]Buried  hydrophobic  residues  normalized  score  at  the  range  [0  to  10].

(b) Consider buried hydrophobic residues

(5)  CONFIDENCE  SCORE=    Buried  hydrophobic  residues  normalized  score  at  the  range  [0  to  10]Solvent  exposed  hydrophobic  residues  normalized  score  at  the  range  [0  to  10].

Hydrophobic cluster analysis is based on a two-dimensional representation of the protein sequence, in which hydrophobic amino acids congregate into clusters [27, 28]. There is a need to assign the confidence score based on conservation score and knowledge of flanking region of hydrophobic residues. A confidence score has been assigned for each residue in the test protein. If the value of confidence score is more than or equal to 1, then residue is highly conserved and if the confidence score is less than 1, then the residue is variable (not well conserved evolutionarily) (Table 2).

**Figure 5:**
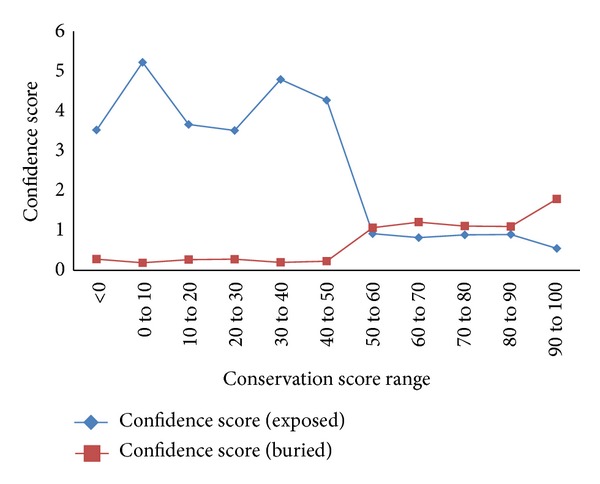
It represents confidence score versus conservation score range.

Buried hydrophobic residues started to dominate while their confidence score was 2.07 at the range from 60 to 70. Hence, it can be concluded that the residue of the query protein is solvent exposed when it obtains a confidence score above 2.07 and the residues are buried hydrophobic residues if the value is below. (Table 2) (Figure 5).

## 4. Case Study

### 4.1. Results

For case study analysis, 10 proteins have been taken randomly from PDB which have chain length of around 300 residues. We assigned the confidence score based on query's homologous sequence to be buried and solvent exposed. After assigning the confidence score, we checked out accuracy of results based on its observed result from NACCESS server which is based on PDB structural results. Over 76% of expected results were accurate, after comparing result from case study proteins with its respective RSA value from NACCESS server.

### 4.2. Case Study Examples Representation Using Pymol Tool

**Figure 6:**
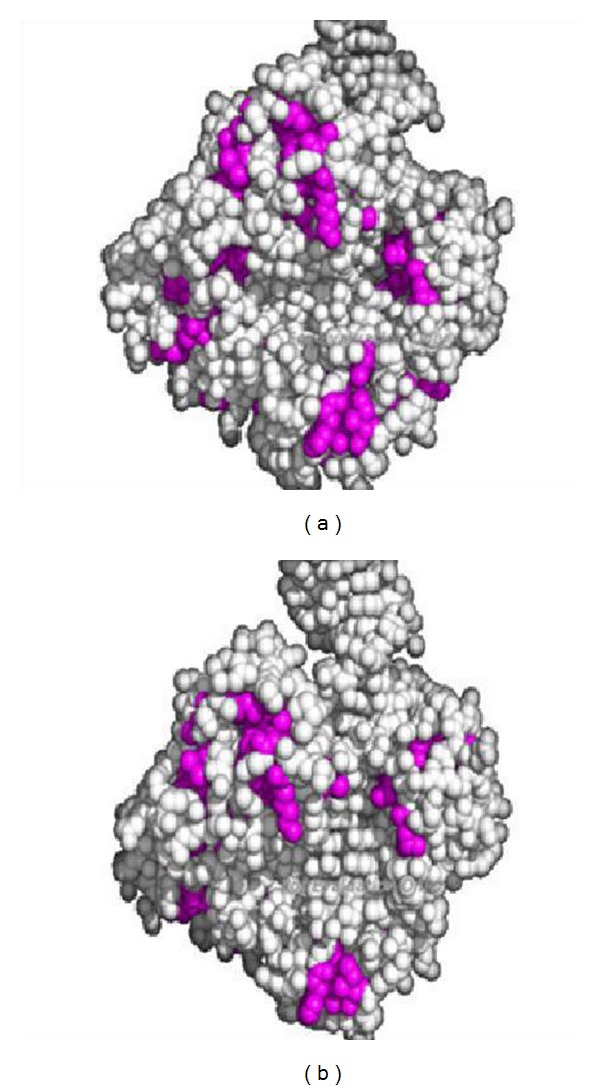
(a) 2UVW-*Sulfolobus solfataricus* P2 DNA polymerase IV (DPO4) (observed). (b) 2UVW-*Sulfolobus solfataricus* P2 DNA polymerase IV (DPO4) (predicted).

Out of these 10 case studies, one protein has been chosen randomly to represent using Pymol tool [29]. Initially, the surface hydrophobic residues were taken into consideration from the randomly selected proteins (Figures 6(a) and 6(b)).

### 4.3. Accuracy Calculation

From the results obtained through the above case study, there was need for analyzing accuracy results by comparing with observed and predicted results.

Consider

(6) $$\begin{array}{c} \text{accuracy}=\frac{\text{number}\;\text{of}\;\text{true}\;\text{positives}+\text{number}\;\text{of}\;\text{true}\;\text{negatives}}{\text{number}\;\text{of}\;\text{true}\;\text{positives}+\text{false}\;\text{positives}+\text{false}\;\text{negatives}+\text{true}\;\text{negatives}}. \end{array}$$

In the above formula,

 True positive = exposed hydrophobic residues as exposed
 True negative = buried hydrophobic residue as buried
 False positive = buried hydrophobic residue as exposed
 False negative = exposed hydrophobic residue as buried.

**Figure 7:**
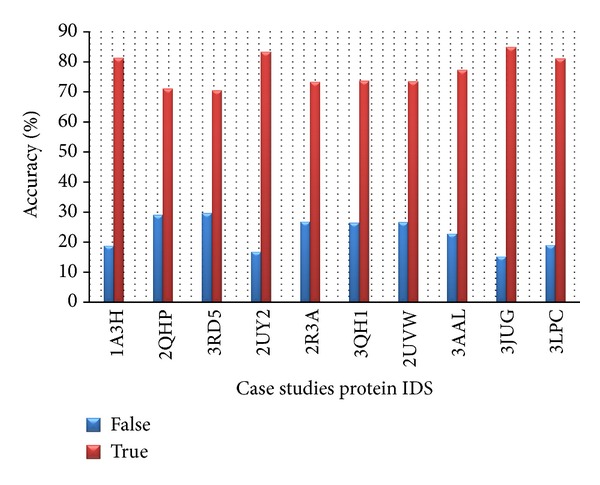
Case studies percentage of accuracy.

After comparing result from case study examples with its respective RSA value from NACCESS server (Figure 7), we have observed that over 76% expected results were accurate. This accuracy has been improved to 78% by implementing knowledge of flanking residues hydrophobic nature.

From Figures 2 and 3, we have analyzed exposed and buried residue propensity in flanking regions (+1, −1) and (+2, −2), respectively and implementation of the following points was obtained to improve the accuracy.

(i) When hydrophobic residues such as Phe, Ile, leu, Met, Val, and Cys occur in flanking regions, hydrophobic propensity values for these residues are considered to be 1. (ii) Hydrophobic propensity value is considered to be 0.75 for the partial hydrophobic residues such as Ala, Tyr, and Trp occurring in flanking regions. (iii) When Ser and Thr occur in flanking regions, the propensity value is considered as 0.35. (iv) When a hydrophilic residue occurs in flanking regions, the propensity value is considered as 0.15.

## 5. Conclusion

Present work is based on nonredundant dataset of monomeric proteins and we have observed that significant 21.4% of hydrophobic residues are solvent exposed which is obtained from RSA analysis information.

After running multiple sequence alignment from the homologous sequences with respect to individual data set proteins, we came to know that exposed hydrophobic residues are poorly conserved and buried hydrophobic residues are highly conserved.

Based on the conservation score of hydrophobic residues obtained from MSA, we assigned confidence score to residues which are likely to be buried and exposed; after comparing the results from 10 proteins and doing a case study with its respective relative surface accessibility value from NACCESS server, we have observed that over 76% expected results were accurate but it has been improved to 78% by considering hydrophobic cluster, that is, flanking residues between +2 and −2 positions.

Knowledge on the solvation state of a residue would be used to identify the solvent exposed hydrophobic residues which can be targeted to increase stability. Hence in the work described here, the approach is adopted in developing a prediction methodology to identify the solvation state of a residue using only the information on sequence. Armed with the knowledge of only monomeric proteins, further research can be carried out to understand behavior of oligomers.

## Supplementary Material

Supplementary file Containing 218 proteins dataset which provides detailed values of no of residues in each protein. No of residues occur on surface and buried & No of hydrophobic residues occur on surface and buried.

## Figures and Tables

**Table 1:** It represents percentage relative frequency of solvent exposed and buried hydrophobic residues in different conservation score range.

Conservation score range	% of relative frequency for solvent exposed hydrophobic residues	% of relative frequency for buried hydrophobic residues
<0	10.68	3.02
0 to 10	4.31	0.82
10 to 20	4.36	1.18
20 to 30	4.87	1.38
30 to 40	5.10	1.06
40 to 50	8.88	2.07
50 to 60	6.70	7.21
60 to 70	6.24	7.56
70 to 80	6.92	7.73
80 to 90	10.73	11.82
90 to 100	31.16	56.07

**Table 2:** Confidence score table.

Conservation score range	% of relative frequency for solvent exposed hydrophobic residues	% of relative frequency for buried hydrophobic residues	Confidence score (exposed)	Confidence score (buried)
<0	10.68	3.02	3.52	0.282
0 to 10	4.31	0.82	5.22	0.19
10 to 20	4.36	1.18	3.66	0.27
20 to 30	4.87	1.38	3.51	0.28
30 to 40	5.10	1.06	4.79	0.20
40 to 50	8.88	2.07	4.27	0.23
50 to 60	6.70	7.21	0.92	1.07
60 to 70	6.24	7.56	0.82	1.21
70 to 80	6.92	7.73	0.89	1.11
80 to 90	10.73	11.82	0.90	1.10
90 to 100	31.16	56.07	0.55	1.79
